# Metabolomic Recovery as a Result of Ischemic Preconditioning Was More Pronounced in Hippocampus than in Cortex That Appeared More Sensitive to Metabolomic Blood Components

**DOI:** 10.3390/metabo11080516

**Published:** 2021-08-05

**Authors:** Eva Baranovicova, Dagmar Kalenska, Marian Grendar, Jan Lehotsky

**Affiliations:** 1 Biomedical Center BioMed, Jessenius Faculty of Medicine, Comenius University in Bratislava, Mala Hora 4, 036 01 Martin, Slovakia; eva.baranovicova@uniba.sk; 2 Department of Anatomy, Jessenius Faculty of Medicine, Comenius University in Bratislava, Mala Hora 4, 036 01 Martin, Slovakia; dagmar.kalenska@uniba.sk; 3 Biomedical Center BioMed, Bioinformatical Unit, Jessenius Faculty of Medicine, Comenius University in Bratislava, Mala Hora 4, 036 01 Martin, Slovakia; marian.grendar@uniba.sk; 4 Department of Medical Biochemistry, Jessenius Faculty of Medicine, Comenius University in Bratislava, Mala Hora 4, 036 01 Martin, Slovakia

**Keywords:** NMR metabolomics, rat, cerebral cortex, hippocampus, heart, blood plasma, ischemia, ischemic preconditioning

## Abstract

The study of an organism’s response to ischemia at different levels is essential to understand the mechanism of the injury as well as protection. We used the occlusion of four vessels as an animal model of global cerebral ischemia to investigate metabolic alterations in cerebral cortex, hippocampus, blood plasma, as well as in a remote organ, the heart, in rats undergoing 24 h postischemic reperfusion. By inducing sublethal ischemic stimuli, we focused on endogenous phenomena known as ischemic tolerance that is currently the best known and most effective way of protecting against ischemic injury. NMR spectroscopy was used to analyze relative metabolite levels in homogenates from rats’ cerebral cortex, hippocampus, and heart together with deproteinized blood plasma. In individual animals subjected to global cerebral ischemia, relative concentrations of the essential amino acids isoleucine, valine, phenylalanine, and tyrosine in cerebral cortex correlated with those in blood plasma (*p* < 0.05, or boundary significant *p* < 0.09). This did not apply for the hippocampus, suggesting a closer relation between ischemic cortex and metabolomic blood components. Hippocampal non-participation on correlation with blood components may emphasize the observed partial or full normalization the post-ischemically altered levels of a number of metabolites in the preconditioned animals. Remarkably, that was observed for cortex to a lesser extent. As a response to the global cerebral ischemia in heart tissue, we observed decreased glutamate and increased 3-hydroxybutyrate. Ischemically induced semi-ketotic state and other changes found in blood plasma partially normalized when ischemic preconditioning was introduced. Some metabolomic changes were so strong that even individual metabolites were able to differentiate between ischemic, ischemically preconditioned, and control brain tissues.

## 1. Introduction

Cerebral ischemic injury is one of the most studied processes due to the high incidence/prevalence in the human population in its different forms. It leads to the cascade of biochemical events that are not always linear, but often circular, sequential, or causal. The re-establishment of blood flow is essential to rescue ischemized tissues. Paradoxically, reperfusion itself results in further damage known as ischemic-reperfusion injury, affecting function and viability of the affected organ [1,2]. As the protection from ever present danger of ischemic injury, living organisms are able to develop a potent protective strategy called ischemic tolerance, where the adaptation to sub lethal stimuli can induce some level of resistance against next ischemic attack [3,4]. Although the exact mechanism is still unknown, the ischemic preconditioning is one of the most effective methods of protection against ischemic injury in both experimental and partially clinical studies [3,5,6].

The experimental model of global cerebral ischemia by four vessels occlusion (4VO) developed by Pulsinelli [7] presents several experimental advantages which included: ease of preparation, a high rate of predictable ischemic neuronal damage, a low incidence of seizures and the absence of anaesthesia for the duration of the ischemic insult [8]. The molecular response to global cerebral ischemia is region-specific due to the different subsets of neurons with different susceptibility and tolerance pattern/level [9,10]. The proteomic and histological changes are in general convincingly recognizable in more than 24 h of postischemic reperfusion [10,11], however delayed neuronal death of hippocampal CA1 pyramidal neurons is detectable several days after the ischemic event [12]. In contrast to these facts, particular metabolomic changes evolve immediately after ischemic insult and progress and sustain over days. Low molecular metabolites, besides being a part of basal biochemical pathways, influence additional molecular processes and signalling pathways [13,14], such as histone acetylation or DNA methylation [15,16], and thus may contribute to further postischemic alterations.

Metabolomic changes in rodent models of cerebral ischemia in brain regions were intensively studied by in vivo [17,18,19] as well as in vitro NMR techniques [20,21]. Ischemic brain injury causes altered metabolomic profiles not only in brain regions but also in the peripheral blood circulation, as shown in the study by Koch et al. [22] where middle cerebral artery occlusion (MCAO) led to hepatic ketogenesis in mice. Similarly, an increase in plasma ketone bodies was observed after global cerebral ischemia in rats [23,24], which, as shown in the next longitudinal study, was most pronounced in 24 h reperfusion time [25]. In the same studies, the protective effect of ischemic preconditioning on plasma metabolites was demonstrated by: (i) a lower extent of observed metabolomic changes [23,24,25] and (ii) faster tendency of plasma metabolites to normalize in preconditioned rats [23,24,25].

Paradoxically, relatively little is known about the metabolomic effect of ischemic preconditioning after global cerebral ischemia in affected brain regions and especially in cerebrally remote organs. The present work was designed to identify metabolomic changes in 24 h postischemic reperfusion in particularly sensitive brain regions, such as:

(i) cerebral cortex
(ii) hippocampus,
(iii) blood plasma, as well as,
(iv) heart as brain’s remote organ, due to its close cerebro-cardial connections.

We also attempted to show the protective effect of ischemic preconditioning on the metabolomic level. In order to identify potential metabolomic biomarkers of ischemic injury and/or ischemic preconditioning both in the cerebral tissues as well as in blood plasma and heart, we employed random forest discrimination algorithm to create ROC (receiver operating characteristic) curve. Lastly, we briefly discuss the influence of different courses of surgery, where the individuality of animals reflected by the level of completeness of ischemia seems to be an important factor affecting the obtained results.

## 2. Results

From all evaluated metabolites (Supplementary Table S1) in particular samples, only metabolites with changes in relative concentrations which are referred as significant (*p* value < 0.05) are listed. Rats with ischemic preconditioning (IPC) showed smaller number of significantly changed metabolites against controls, and the extent of the changes was, generally, lower. The results for cerebral cortex, hippocampus, heart, and blood are listed in Table 1. The quantitatively evaluated metabolites that did not show significant changes between groups, were:

In cortex: succinate, alanine, aspartate, myo-inositol, creatine, taurine, ascorbate.
In hippocampus: isoleucine, valine, alanine, glutamine, phenylalanine, fumarate.
In heart: isoleucine, valine, alanine, glutamine, succinate, creatine, taurine, phenylalanine, tyrosine, fumarate, inosine.
In blood plasma: tyrosine.

Relative concentrations of four essential amino acids in blood plasma and brain tissue extracts were correlated by Pearson’s correlation, where significant, or boundary significant correlations were found for cortex and blood plasma in IR animals. Related R score and *p* values are summarised in Table 2. Similar correlation for IPC rats was not performed as based on differences in the direction of metabolic changes for alanine and tyrosine in blood and brain tissues whose correlation cannot be assumed.

**Figure 1:**
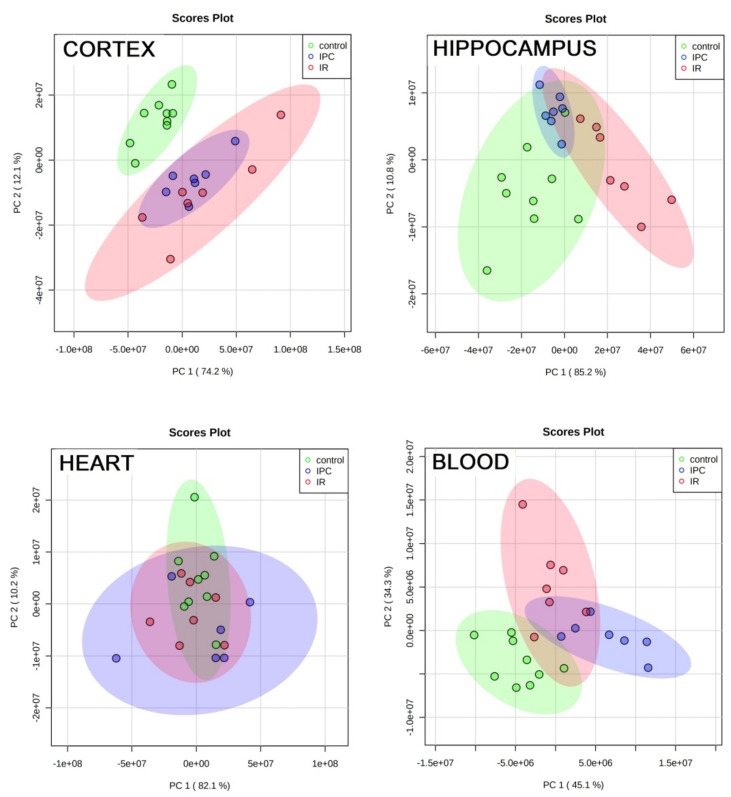
PCA analyses of system control-IR-IPC for cortex, hippocampus, heart and blood plasma in rats, as variables were used relative concentrations of metabolites determined by NMR.

PCA analysis serves the 2D visualization of multidimensional data and, from the diagrams, data proximity can be estimated. As shown in Figure 1, there are notable differences among measured metabolites levels in tissue extracts and blood plasma in C, IR and IPC rats. Since in cortex, the IR and IPC rats are well separated from controls, in hippocampal tissue extracts C and IPC rats overlap and IR rats deviate from them. The heart tissue extract shows somewhat similar metabolomic features in C, IR, and IPC animals. The PCA diagram from blood plasma indicates metabolomic differences among all three groups.

In addition to PCA, we run also PLS-DA analyses, including discriminatory algorithm (Supplementary Figure S1). These showed the promising successful discrimination between controls, IR and IPC rats in cortex, hippocampus, and blood plasma. The leave one out cross-validated PLS-DA performed very promising, however in some cases the algorithm performed with negative Q2 value, what is a sign of over-fitted model (for details, please see Supplementary Table S2). The discrimination potential of systems in the context of potential biomarkers was investigated by Monte Carlo cross-validated Random Forest discrimination algorithm using balanced subsampling, that is, unlike PLS-DA, known to be robust against overfitting and outliers [26]. Similarly to PCA and PLS-DA, relative concentrations of metabolites were used as variables. Discriminatory power is expressed in AUC values derived from ROC curve (for more details [27]). We chose an approach where we calculated AUC for individual metabolites (Table 3). By the combination of variables showing the highest AUC, almost in all cases, the ideal discrimination with AUC of 1 was achieved.

## 3. Discussion

### 3.1. Cerebral Cortex and Hippocampus

#### 3.1.1. Neurotransmitters and Related Metabolites

**Figure 2:**
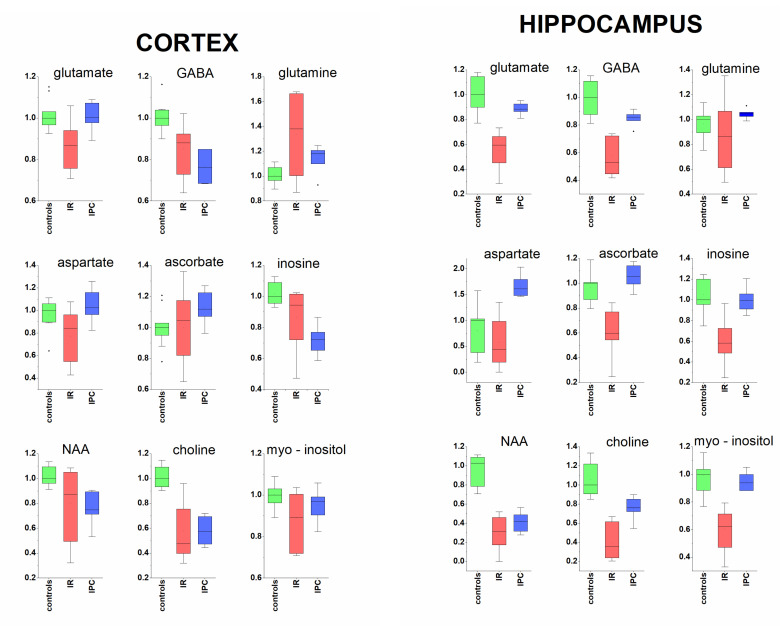
Metabolomic response of cortex (**left**) and hippocampus (**right**) to 24 h reperfusion after global cerebral ischemia, with and without ischemic preconditioning. Relative amounts of metabolites in tissue extracts were determined by 1H NMR, scaled to median of controls set = 1, IR—ischemia reperfusion, IPC—ischemic preconditioning.

Hippocampal and cortical tissue manifested particular similarities as well as differences in metabolomic response to global cerebral ischemia in 24 h postischemic reperfusion (Table 1, Figure 2). Glutamate acts as one of the most important neurotransmitters for normal brain function as well as the major effector of cell damage after global ischemia [28,29], where in ischemic brain extracellular glutamate is elevated rapidly after the onset of ischemia and declines following reperfusion [30]. In time of 24 h after global cerebral ischemia, we observed significantly decreased glutamate content in cortical as well as in hippocampal tissue related in tissue weight, similarly as it was described by Hu et al. in tissue extract from ischemic cerebral hemisphere after MCAO [21], as well as by Zhang et al. in mice after global cerebral ischemia in both cortex and hippocampus [20]. In contrast, strong regional specific analysis showed increased glutamate level, however calculated relatively to protein content, in 24 h of postischemic reperfusion in the ischemic core (striatum and overlying cortex) and penumbra (adjacent cortex) in MCAO model [31]. In the same study, rapid remote ischemic preconditioning normalized the glutamate level. The protective effect of ischemic tolerance induced by sub-lethal ischemic stimuli used in this study was, in both, cortex and hippocampus manifested in the restoration of post-ischemically decreased glutamate level to the level of control animals.

The relative tissue amounts of inhibitory neurotransmitter GABA were significantly decreased in rats’ hippocampus and cortex in 24 h reperfusion, and, similarly to glutamate, the change was more pronounced in hippocampus. This finding is in accordance with recent studies that showed, in animals [32,33] and humans [34] suffering ischemic attacks, that GABA contents in the affected cerebral hemisphere actually declined. Ischemic preconditioning (IPC) was manifested by the restoration of GABA level to the level of control animals in hippocampus, but not in the cortex.

The neurotransmitters glutamate and GABA are metabolically interconnected with glutamine in glutamine-glutamate (GABA) cycle [35]. The relative glutamine content in tissues after 24 h reperfusion was increased in cortex, but without significant change in hippocampus. The alterations in tissue content of glutamine, may influence also inflammatory response following ischemic attack that contribute to damage as well as to tissue repair [36], since glutamine besides other functions serves as a fuel for immune cells, i.e., lymphocytes, neutrophils, and macrophages, and also plays a crucial role in the cytokine production [37]. Aspartate acts as an important metabolite in brain, including glutamate metabolism, however, its supposed function as a neurotransmitter was excluded in the study by Herring et al. [38]. Its decrease in rats’ cortex after cerebral ischemia was not followed in hippocampus, where, interestingly, its relative amount in IPC rats obviously exceeded the level of control animals. Taking together, global cerebral ischemia affected not only neurotransmitters levels in the brain tissues, but also naturally affected the levels of their metabolomic co-partners.

#### 3.1.2. Acorbate as Antioxidant

The brain, being an organ intensively metabolizing oxygen and having relatively weak protective antioxidant mechanisms, is particularly susceptible to oxidative stress, that is one of the key factors initiating IR injury [39]. To maintain redox balance, the brain regions depends on high levels of antioxidants, and the most abundant antioxidant present in brain tissue is ascorbate. Just ascorbate radicals were increased in hypoperfuzed aged rat brains [40] confirming neuroprotective role of ascorbate as an effective radical scavenger in oxidation stress. Unlike humans, rats have the ability to synthetize vitamin C in liver and release it into the circulation. In our study, we observed strong depletion of ascorbate pool in the hippocampus in IR rats. However, rats with ischemic preconditioning managed to sustain ascorbate accumulation in hippocampus to the control level in 24 h postischemic reperfusion. The pattern differs in cortex, where the increased ascorbate levels in IPC rats suggest ascorbate gathering in cortical tissue.

#### 3.1.3. Metabolites Known from In Vivo MRS

NAA is considered to be a marker of neuronal viability/or health, since it is almost exclusive localized in neurons [41]. After transient ischemia and brain injury without neuronal death, as well as after long-term focal cerebral ischemia, attenuated NAA levels are able to recover [42,43], suggesting its role as a marker of neuronal functionality rather than neuronal density. Significantly decreased NAA levels in both brain tissue extracts imply for persistent disturbed condition of neurons present also in ischemic preconditioned animals. Related to the observed percentage change of NAA level, the postischemic alterations occurred to a much greater extent in hippocampal than in cortical neurons.

Myo-inositol is an organic osmolyte promoting cell membrane stability with function as effective radical scavenger [44] and potential to provide neuroprotection during/following stroke [45]. It was documented to be decreased in the ischemized cerebral hemisphere in 24 h after MCAO [21]. In 24 h reperfusion after global cerebral ischemia, we observed only slight, not-significant decrease of myo-inositol in cortex, but statistically significant decrease in hippocampus. In IPC rats, the myo-inositol concentrations were found to be close to the control level in both cerebrocortical and hippocampal tissue extracts (Figure 2).

In our study, we observed significantly decreased choline levels in both, rats cortical and hippocampal tissue extracts in 24 h reperfusion (Figure 2). Many animals can synthesize choline de novo, but the production is often insufficient, and choline must be supplemented by diet. Choline conferred brain protection against ischemic stroke in rats [46] and the other study showed the neuroprotective effect of oral choline administration after global brain ischemia in rats [47]. The availability of choline is a rate-limiting factor in phospholipid synthesis and, therefore, it may be important for timely membrane repair and cell survival.

### 3.2. Protective Effect of Ischemic Preconditioning in Brain Tissue Extracts

Animals subjected to sub-lethal ischemic preconditioning prior to 15 min. global cerebral ischemia showed lower extent of observed postischemic changes in a number of metabolites in both tissue extracts. This is in line of partially protected cerebral histo-morphological patterns [3,24,25]. In hippocampus, the relative concentrations of almost all metabolites responded by full or partial restoration, when IPC was introduced. Besides metabolites shown in Figure 2, this trend was observed also for to creatine, taurine and succinate (Table 1). The notable exceptions in hippocampal tissue were NAA and aspartate. In cerebral cortex, besides already discussed metabolites, the restoration of isoleucine, valine, alanine, phenylalanine, and fumarate (Table 1) in IPC rats was observed.

### 3.3. Heart and Blood Plasma

#### 3.3.1. Glutamate in Heart Tissue

**Figure 3:**
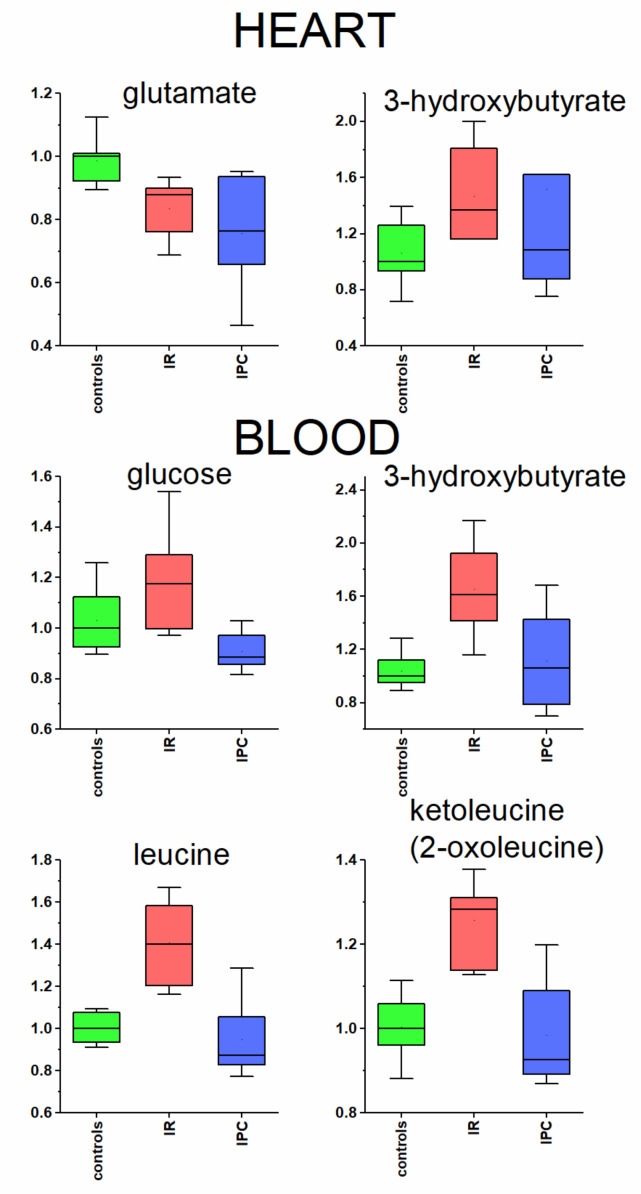
Relative changes in metabolites levels in heart tissue extracts and blood plasma, determined by 1H NMR, scaled to median of controls set = 1, IR—ischemia reperfusion, IPC—ischemic preconditioning.

Brain ischemia induces changes not only in the affected tissue, but also evokes systemic changes in distinct organs [48], including blood plasma. Heart due to its close cerebrocardial connections might be influenced by cerebral ischemic damage also on the metabolomic level. In 24 h reperfusion after global cerebral ischemia, we observed significant changes in heart tissue extract for two metabolites. First, glutamate was significantly decreased in the heart of the ischemic rat, without any observable protective IPC effect, whereas the glutamate level remained lower also in preconditioned rats (Table 1, Figure 3). Glutamate can be used in energy metabolism as a substrate for alpha-ketoglutarate, TCA cycle participant. In addition, the proven presence of ionotropic [49] and metabotropic [50] glutamate receptors in rat heart suggests that cardiac function may be affected by cerebral ischemic event.

#### 3.3.2. Metabolites Participating in Energy Metabolism

The second metabolite significantly changed in heart tissue extract as well as in blood plasma in IR animals was 3- hydroxybutyrate (Table 1, Figure 3). Based on the metabolomic changes found in the blood plasma after global cerebral ischemia in this as well as in previous studies [22,23,24,25], the energy metabolism is shifted towards accelerated synthesis of ketone bodies. The switch-over of heart tissue to increased utilization of ketone bodies in the stress condition, including cerebral ischemia, has been proven in many studies [51,52]. Besides fulfilling the needs of heart, ketones can also provide as much as 70% of the brain’s energy needs more efficiently than glucose [53]. When 3-hydroxybutyrate is used as an energy substrate in partially ischemic brain, it has protective effects on cerebral hypoxia, anoxia, and ischemia-induced metabolic changes [54]. The increase in blood glucose in IR rats, together with decrease in its metabolic products indicates deprived glycolysis. The hyperglycaemia is often accompanied by increase in plasma levels of branched chain amino acids (BCAAs) [55], in accordance with results presented in our study. In ischemically preconditioned rats, the 3-hydroxybutyrate level in heart tissue as well as in blood plasma normalized to control level (Figure 3). Besides that, the broad group of plasma metabolites (glucose, BCAAs, BCKAs) were also normalized in 24 h postischemic reperfusion in IPC rats in blood plasma. In Figure 3, only leucine and ketoleucine are shown as the trend for valine and isoleucine and their ketoacids was very similar to those for leucine and ketoleucine. Taking together, the IPC rats showed the ability to normalize disturbed energy metabolism in 24 h postischemic period. The overall metabolomic changes in blood plasma found in this study are listed in Table 1, and the detailed discussion can be derived from our previous works [23,24,25]. It is important to note that, after cerebral brain ischemia, all organs are indirectly affected by challenging the alterations in metabolomic plasma composition.

### 3.4. Brain and Plasma Metabolites—Correlation

The brain is protected from the fluctuation of plasma metabolites by a blood-brain barrier (BBB) to sustain an optimal chemical environment for cerebral function. BBB disruption appears shortly after the onset of cerebral artery occlusion and lasts for days, changing the extent of BBB permeability alterations over time [56]. The increased BBB permeability due to BBB disruption after cerebral ischemia was confirmed in many experiments, e.g., by injecting tracers [57] or dye [31] intravenously into stroke animals.

Since the tissues and blood plasma in our study originated from the same individuals, we run statistical tests to examine the correlation between the relative concentrations for essential amino acids, namely isoleucine, valine, phenylalanine, and tyrosine, in blood plasma and in brain tissues in controls and in rats after global cerebral ischemia. The first and the third column in Table 2 showed very similar trends in metabolites profile between cortex and plasma in IR rats against controls. For IR rats, statistically significant or boundary significant correlation were found for all four evaluated amino acids between cerebral cortex and blood plasma. Interestingly, no signs of analogous correlation were found for hippocampal tissue extract and blood plasma, and no similar correlations were found in control animals. This implies for particularly tighter communication of the ischemized cortex to metabolic blood components, which is not manifested, based on presented results, for hippocampus. One of the possible explanations comes from the larger communicative volume of affected cortical layers compared to the tiny hippocampal region with the blood plasma.

The differences in diffusion of small metabolites through ischemically challenged BBB to cortical and hippocampal tissues may partially contribute to metabolic recovery after ischemia. Based on our results, the hippocampus seems to be involved to a lesser manner on direct (diffuse) small metabolites transfer from blood and its response to ischemia may be rather regionally controlled. On the other hand, the ischemic cortex is, due to the found correlation, more highly challenged with altered metabolomic blood composition, which might influence the recovery after ischemia and impact the normalization of metabolites in IPC rats. These findings may be a part of the reflected region-specific response to cerebral ischemia, as known from previous studies [9,10].

### 3.5. PCA and Groups’ Proximity

PCA analyses visualized the above discussed findings in other way. The ischemic insult caused differentiation of tissues metabolic content in cortex and hippocampus (IR against C). The effect of ischemic preconditioning, more pronounced in hippocampus (Table 1), was reflected in the overlap of C and IPC rats in hippocampus, but not in cortex. Rather, metabolomic proximity is expected in heart tissue extracts in 24 h reperfusion after 4VO, as confirmed by Dun’s post hoc test where only two metabolites were significantly changed. Plasma metabolomes are relatively separated in C, IR, and IPC rats, as already discussed in our previous works [24,25] in detail.

### 3.6. Random Forest towards Biomarkers

In our previous studies we showed a high potential of plasma metabolites to serve as potential biomarkers of cerebral brain ischemia in rats [24,25]. In the cortical and hippocampal tissues, there were even individual metabolites identified that are able to discriminate with very high sensitivity/specificity achieving AUC values of 1 or very close to 1 (Table 3), interestingly not the same. The discrimination becomes more robust when metabolites with reported high discrimination power work in combination. Based on results in Table 3, there were also metabolites found that may serve as discrimination agents regarding ischemic preconditioning, and again, they differ for cortex and hippocampus. In heart tissue, glutamate may be indicative molecule (AUC near 0.90) of ischemic injury on the brain, however disregarding ischemic preconditioning (Table 3).

### 3.7. Methodological Note to Four Vessels Occlusion

It is impossible to achieve a complete stop of blood flow in all animals during 4VO due to the individual differences in vein anatomy, as described by Pulsinelli [7]. The levels of plasma metabolites, metabolites evaluated in cerebral cortex, hippocampus, and heart in animals after detected and verified incomplete ischemia ranged between levels of control and IR animals (see Supplementary Figure S2). This finding is not surprising. However, it confirms that the extent of metabolomic changes is strongly related to ischemia severity and provides support for the precious ischemic protocol.

## 4. Materials and Methods

### 4.1. Induction of Ischemic Preconditioning and Ischemia

Male Wistar rats at the age of 4 months (having a mean weight of 383 ± 46 g) were used. From a total amount of 40, nine controls (C), seven rats with ischemia (IR), and seven ischemically preconditioned rats (IPC) were included in further evaluation. The complete ischemia was not achieved in eight animals, and nine animals died during or after surgery. Rats were housed in a temperature-controlled room 22 ± 2 °C on a 12-h light/dark cycle with free access to food and water.

**Figure 4:**
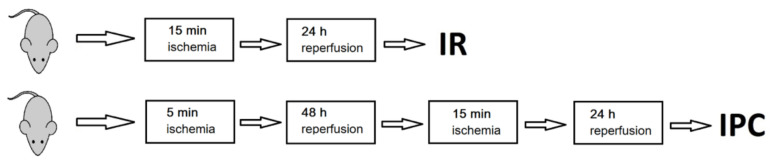
Schematic representation of operating steps in rats, IR—rats with 24 h postischemic reperfusion, IPC—ischemically preconditioned rats with 24 h postischemic reperfusion.

Global cerebral ischemia was induced by four vessels occlusion model as introduced by Pulsinelli [7] and already described in previous papers [24,25]. On day 1, bilateral vertebral arteries were irreversibly electro-cauterized under anaesthesia with sevoflurane (a mixture of 3.5% sevoflurane in 33% O_2_ and 66% N_2_O). Ischemia: on the second day, animals underwent 15 min ischemia. After 24 h reperfusion, animals were sacrificed. Ischemic preconditioning and ischemia: on the second day, animals were anaesthetised to gently dissect both common carotid arteries. Ischemia was induced by occluding the arteries on awakened animals for 5 min to induce sub-lethal ischemia. Then, 48 h later, both common carotids were occluded to induce 15 min ischemia. After 24 h of reperfusion, animals were sacrificed (see Figure 4 for schematic representation).

Only rats that lost their righting reflex were unresponsive and whose pupils were dilated during ischemia were selected to evaluate complete cerebral ischemia, as recommended by Pulsinelli [58]. Rats that did not meet the conditions of ischemia completeness were used to describe the impact of ischemia incompleteness.

### 4.2. Organ Collection and Blood Plasma Samples Preparation

After decapitation, organs were immediately removed while placed on ice, stored at −80 °C. Before freezing, the heart was washed out with saline through the aorta. Tissues were homogenized still in the frozen state, where the ratio of 1g tissue to 5ml ACN/H2O (1/1, vol.) was kept for cortex, hippocampus, and the heart as well. For cortex and hippocampus, Potter’s homogenizer (1100 rpm, 1 min) and for heart, POLYTRON PT 1600E homogenizer (9500 rpm, 1 min) were used. After centrifugation at 4 °C, 10,625× *g*, 10 min, 600 µL of supernatant from cortex and heart and 400 µL of supernatant from hippocampus were dried out and stored at −80 °C until measurements.

Blood was collected in heparin-coated tubes, centrifuged at 4 °C, 2000 rpm, for 20 min. Plasma was frozen at −80 °C until used. After decapitation, we obtained 4–6 mL of blood from a single animal leading to less than half volumes of plasma. Plasma deproteination was carried according to Gowda et al. [59] by adding 600 µL of Methanol to 300 µL of plasma. After vortexing, the mixture was stored at −20 °C for 20 min, and then centrifuged at room temperature for 15 min at 14,462× *g*. Then, 650 µL of supernatant were dried out. Stock solution consisted of phosphate buffer 100 mM and 0.30 mM TSP-d_4_ (trimethylsilylpropionic acid-d_4_) as a chemical shift reference in deuterated water. For measurements, we carefully mixed dried materials with 100 µL of stock solutions and 500 µL of deuterated water (98.9%). Then, 550 µL of final mixture were transferred into 5 mm NMR tube.

### 4.3. NMR Data Acquisition

NMR data were acquired on 600 MHz NMR spectrometer Avance III from Bruker equipped with TCI cryoprobe at T = 310 K. Initial settings (basal shimming, receiver gain, water suppression frequency) were done on an independent sample and adopted for measurements. After preparation, samples were stored in a Sample Jet automatic machine, cooled at approximately 5 °C. Before measurement, each sample was preheated to the 310 K for 5 min. An exponential noise filter was used to introduce 0.3 Hz line broadening before Fourier transform. All data were zero filled once. Samples were randomly ordered for acquisition.

### 4.4. Data Acquisition, Processing and Evaluation

We modified standard profiling protocols from Bruker as follows: noesy with presaturation (noesygppr1d): FID size 64 k, dummy scans 4, number of scans: 512 for tissues and 128 for blood plasma, spectral width 20.4750 ppm; cosy with presaturation (cosygpprqf): FID size 4 k, dummy scans 8, number of scans 1, spectral width 16.0125 ppm; homonuclear J- resolved (jresgpprqf): FID size 8 k, dummy scans 16, number of scans 4; profiling cpmg (cpmgpr1d, L4 = 126, d20 = 3ms): FID size 64 k, dummy scans 4, number of scans 64 for tissues and 128 for blood, spectral width 20.0156 ppm. All experiments were conducted with a relaxation delay of 4 s. Spectra were solved using human metabolomic database (Available online: www.hmdb.ca (accessed on January 2021)) [60], chenomix software free trial version, internal metabolite database and researching in metabolomic literature [59]. The proton NMR chemical shifts are reported relative to TSP-d_4_ signal, which was assigned a chemical shift of 0.000 ppm. The peak multiplicities were confirmed in J-resolved spectra and homonuclear cross peaks were confirmed in 2D cosy spectra. Peak assignments are listed in Supplementary Table S1.

All spectra were binned to bins of the size of 0.001 ppm (Amix, Bruker). No additional normalisation method was applied on the data. For evaluation, data from cpmg acquisition were used. We summed intensities of selected bins for spectra subregions with only one metabolite assigned or minimally affected by other co-metabolites. Metabolites showing weak intensive peaks or substantial peak overlap were excluded from the evaluation. The obtained values were used as relative concentrations of particular metabolites related to weight for tissues and to volume for blood plasma.

### 4.5. Statistics

The data were explored and analysed in Matlab 2018b, Origin Pro 2019 and metaboanalyst [29]. Box plots were used to explore scaled metabolite intensities. The null hypothesis of equality of population medians in controls, IR and IPC rats was tested by the non-parametrical Kruskal–Wallis test with Dun’s post hoc test for pair wise comparison. Fold-change was computed using the median of intensities as the difference of the medians divided by the median of the comparison group. PCA and PLS-DA analyses were calculated in metabo-analyst 5.0 [61]. To measure a correlation between two sets of data, namely relative concentration in tissue extracts and in blood plasma, Pearson’s correlation was used. For discriminatory analyses, we employed a cross-validated random forest discriminatory algorithm to obtain AUC values derived from ROC curves.

## 5. Conclusions

Metabolomic changes in in 24 h reperfusion after global cerebral ischemia were observed beside others for neurotransmitters glutamate and GABA and naturally for their co-metabolites, for ascorbate as the most abundant radical scavenger and for NAA as a indicator of neuronal health. Cerebral cortex and hippocampus showed similar features, such as decreased levels in glutamate, GABA, inosine, NAA, choline, and myoinositol, remarkably more pronounced in hippocampal tissue extracts. The differences in tissues response to ischemia were manifested in disparity in ascorbate, taurine, fumarate, tyrosine, and alanine levels. Ischemic preconditioning was presented in partially or fully restoration of post-ischemically reduced metabolite levels, what was predominately observed in hippocampal tissue extract. The relative levels of essential amino acids isoleucine, valine, phenylalanine, and tyrosine in ischemized individuals correlated significantly or were boundary significant between cortical tissue extract and blood plasma. This relation was observed neither for hippocampal tissue nor for control animals. These findings may reflect the region-specific response to cerebral ischemia, as, based on our results, the response of hippocampus might be rather regionally controlled. On the other hand, due to found correlation, postischemic recovery of cortex may be challenged with metabolomic blood composition.

The metabolomic changes in the heart showed decreased glutamate and increased 3-hydroxybutyrate levels, suggesting potential metabolomic response of the cerebrally distant organ to global ischemia. Analysis of blood plasma demonstrated that the ischemically preconditioned rats had the ability to partially normalize altered energy metabolism, postischemically shifted towards accelerated ketone bodies synthesis.

In addition, certain metabolic changes were so strong that metabolites alone (such as GABA, NAA, inosine, choline, tyrosine, ascorbate, and others) might serve as potential biomarkers of ischemic injury, although the metabolites of the highest discriminatory power were partly different for cortical and hippocampal tissue extracts. Similarly, the potential biomarkers of ischemic preconditioning were different for both evaluated brain regions. Lastly, we briefly demonstrated the influence of anatomically derived ischemic incompleteness to the extent of observed metabolomic response in individual tissues and blood plasma, which highlights the need for the precise ischemic protocol.

## Figures and Tables

**Table 1:** Statistical evaluation of changes in relative concentrations of metabolites in particular rat tissues and blood plasma in 24 h reperfusion after 4VO, with and without ischemic preconditioning (IR—24 h reperfusion, IPC—ischemic preconditioning + 24 h reperfusion, C—control animals, FC—fold change, n.s. not significant). *p* values were derived from non-parametric Kruskal—Wallis and Dun post-hoc tests and adjusted by Benjamin-Hochberg correction for multiple testing.

Cortex	Kruskal-Wallis	IR/C		IR/IPC		IPC/C	
	*p* Value	*p* Value	FC	*p* Value	FC	*p* Value	FC
isoleucine	0.042	0.003	0.53	0.027	−0.31	0.423	0.07
valine	0.042	0.003	0.61	0.027	−0.35	0.423	0.04
glutamine	0.009	0.009	0.38	0.223	−0.15	0.126	0.17
glutamate	0.073	0.015	−0.16	0.017	0.19	0.943	0.00
4-aminobutyrate	<0.001	0.015	−0.14	0.503	−0.11	0.001	−0.24
niacineamide	0.009	0.014	−0.15	0.635	0.02	0.022	−0.13
phenylalanine	0.005	0.013	0.64	0.005	−0.41	0.402	−0.04
tyrosine	0.016	0.762	0.03	0.007	−0.22	0.007	−0.20
fumarate	0.042	0.003	0.40	0.039	−0.27	0.345	0.03
inosine	0.003	0.084	−0.06	0.289	−0.24	0.004	−0.28
ascorbate	0.061	0.850	0.03	0.299	0.07	0.282	0.10
NAA	0.003	0.004	−0.16	0.963	−0.12	0.004	−0.25
choline	<0.001	0.001	−0.56	0.413	0.31	0.008	−0.42
**Hippocampus**							
glutamate	0.002	0.000	−0.41	0.032	0.49	0.194	−0.12
4-aminobutyrate	0.002	0.000	−0.47	0.045	0.62	0.123	−0.14
succinate	0.005	0.004	−0.33	0.012	0.37	0.783	−0.08
aspartate	0.016	0.793	−0.49	0.005	2.16	0.005	0.61
myo-inositol	0.005	0.002	−0.38	0.008	0.51	0.709	−0.06
creatine	0.005	0.004	−0.29	0.007	0.44	0.948	0.02
taurine	0.005	0.003	−0.25	0.029	0.31	0.465	−0.02
tyrosine	0.042	0.009	−0.32	0.208	0.28	0.197	−0.13
inosine	0.042	0.004	−0.42	0.028	0.71	0.537	−0.01
ascorbate	0.001	0.005	−0.41	0.001	0.78	0.355	0.06
NAA	0.009	0.008	−0.68	0.612	0.30	0.030	−0.58
choline	*p* < 0.0001	0.000	−0.64	0.085	1.14	0.050	−0.24
**Heart**							
glutamate	0.2	0.008	−0.11	0.830	−0.13	0.008	−0.23
3-hydroxybutyrate	0.055	0.048	0.34	0.253	−0.20	0.413	0.07
**Blood Plasma**							
lactate	0.009	0.004	−0.26	0.766	0.01	0.011	−0.25
alanine	0.001	0.004	−0.34	0.341	−0.10	0.000	−0.40
valine	0.007	0.016	0.28	0.004	−0.33	0.552	−0.15
glucose	0.017	0.123	0.19	0.001	−0.27	0.064	−0.13
leucine	0.007	0.008	0.41	0.004	−0.38	0.746	−0.13
isoleucine	0.006	0.006	0.40	0.003	−0.39	0.771	−0.14
acetate	0.001	0.001	−0.32	0.861	0.08	0.002	−0.27
acetoacetate	0.001	0.000	2.27	0.421	−0.23	0.003	1.53
pyruvate	0.001	0.000	−0.44	0.661	0.07	0.002	−0.40
citrate	0.013	0.005	−0.30	0.811	−0.09	0.004	−0.37
phenylalanine	0.042	0.004	0.31	0.020	−0.21	0.636	0.04
glutamine	0.013	0.084	−0.14	0.369	−0.03	0.018	−0.16
lysine	0.686	0.929	−0.03	0.329	−0.08	0.494	−0.10
3-hydroxybutyrate	0.002	0.003	0.66	0.007	−0.34	0.849	0.09
ketoleucine	0.006	0.003	0.30	0.001	−0.28	0.636	−0.07
ketoisoleucine	0.007	0.008	0.21	0.005	−0.22	0.827	−0.05
ketovaline	0.007	0.009	0.22	0.004	−0.26	0.695	−0.10
tryptophan	0.013	0.027	−0.11	0.464	−0.03	0.006	−0.14

**Table 2:** Visualization of changes in metabolite ´s levels in brain tissue extracts and blood plasma in IR rats against controls (the first three columns), the arrow indicates the direction of change, * statistically significant change. The next two columns -Pearson’s correlation derived R score and *p* value for relative metabolite levels in organs in individual animals, with *p* value adjusted (*p* value adj) by Bejamini-Hochberg correction for multiple data testing, showed for IR rats.

	Cortex IR/Controls	Hippocampus IR/Controls	Blood IR/Controls	Cortex/Blood R Score/*p* Value/ *p* Value Adj (IR Rats)	Hippocampus/Blood R Score/*p* Value/ *p* Value Adj (IR Rats)
isoleucine	↑ *	↑	↑ *	0.7654/0.045/0.089	0.073/0.87/1
valine	↑ *	↑	↑ *	0.9066/0.012/0.048	0.056/0.90/0.9
phenylalanine	↑ *	↑	↑ *	0.7147/0.071/0.071	0.257/0.57/1
tyrosine	no change	↓ *	no change	0.7379/0.058/0.077	−0.246/0.59/1

**Table 3:** The discriminatory power of individual metabolites expressed by AUC value derived from ROC curve after cross validated Random Forest discrimination. Algorithm was fed by relative concentrations of metabolites in particular samples. Only metabolites with AUC > 0.80 are listed.

Sample	Groups	Metabolites with Individual AUC Values
cortex	IR/controls	fumarate 1, choline 0.998, phenylalanine 0.997, valine 0.995, isoleucine 0.925, glutamine 0.923, alanine 0.842, GABA 0.809
cortex	IPC/controls	GABA 1, inosine 1, choline 1, tyrosine 0.996, NAA 0.990, ascorbate 0.943, glutamine 0.804
cortex	IPC/IR	phenylalanine 1, valine 0.995, isoleucine 0.958, fumarate 0.939, tyrosine 0.924, alanine 0.906, ascorbate 0.901, glutamine 0.829
hippocampus	IR/controls	glutamate 1, GABA 1, choline 1, myo-inositol 0.978, ascorbate 0.963, inosine 0.919, NAA 0.918, creatine 0.894, succinate 0.878
hippocampus	IPC/controls	aspartate 0.936, choline 0.912, NAA 0.931
hippocampus	IPC/IR	glutamate 1, myo-inositol 1, ascorbate 1, succinate 0.999, aspartate 0.991, GABA 0.979, creatine 0.997, choline 0.941, inosine 0.913
heart	IR/controls	glutamate 0.898
heart	IPC/controls	glutamate 0.882
heart	IPC/IR	none

## Data Availability

All data is available on request from Eva Baranovicova.

## Supplementary Materials

The following are available online at https://www.mdpi.com/article/10.3390/metabo11080516/s1, Table S1: Chemical shifts (in ppm), J couplings (in Hz) and multiplicities for the pool of metabolites identified in tissue extracts and deproteinated plasma by NMR. The proton NMR chemical shifts are reported relative to TMS-d4 signal which was assigned a chemical shift of 0.000 ppm; + metabolite found, −metabolite not found, or assignment of peaks was not unambiguous. Chemical shifts in particular material may differ from values listed, however the difference did not exceed 0.003 ppm, Figure S1: PLD-DA analyses of system C-IR-IPC for cortex, hippocampus, heart, and blood plasma in control, IR, and IPC rats, as variables were used relative concentrations of metabolites determined by NMR, Table S2: Results from leave-one-out cross-validated PLS-DA statistical discrimination: R2, Q2 performance measured by accuracy, * negative Q2 is a sign of overfitted model, Figure S2: Relative levels of selected plasma metabolites in cortex, hippocampus, and heart in animals after detected and verified incomplete ischemia, *-rats not fulfilling criteria for ischemia completeness, IR-rats fulfilling criteria for ischemia completeness.
