# Supplementary material for: Metabolomic Recovery as a Result of Ischemic Preconditioning Was More Pronounced in Hippocampus than in Cortex That Appeared More Sensitive to Metabolomic Blood Components
Source: Metabolites. 2021 Aug 5;11(8):516. doi: 10.3390/metabo11080516 (PMC8398863; doi:10.3390/metabo11080516)
Supplement: Supplementary file 1 [file metabolites-11-00516-s001.zip › metabolites-1293447-supplementary.pdf]

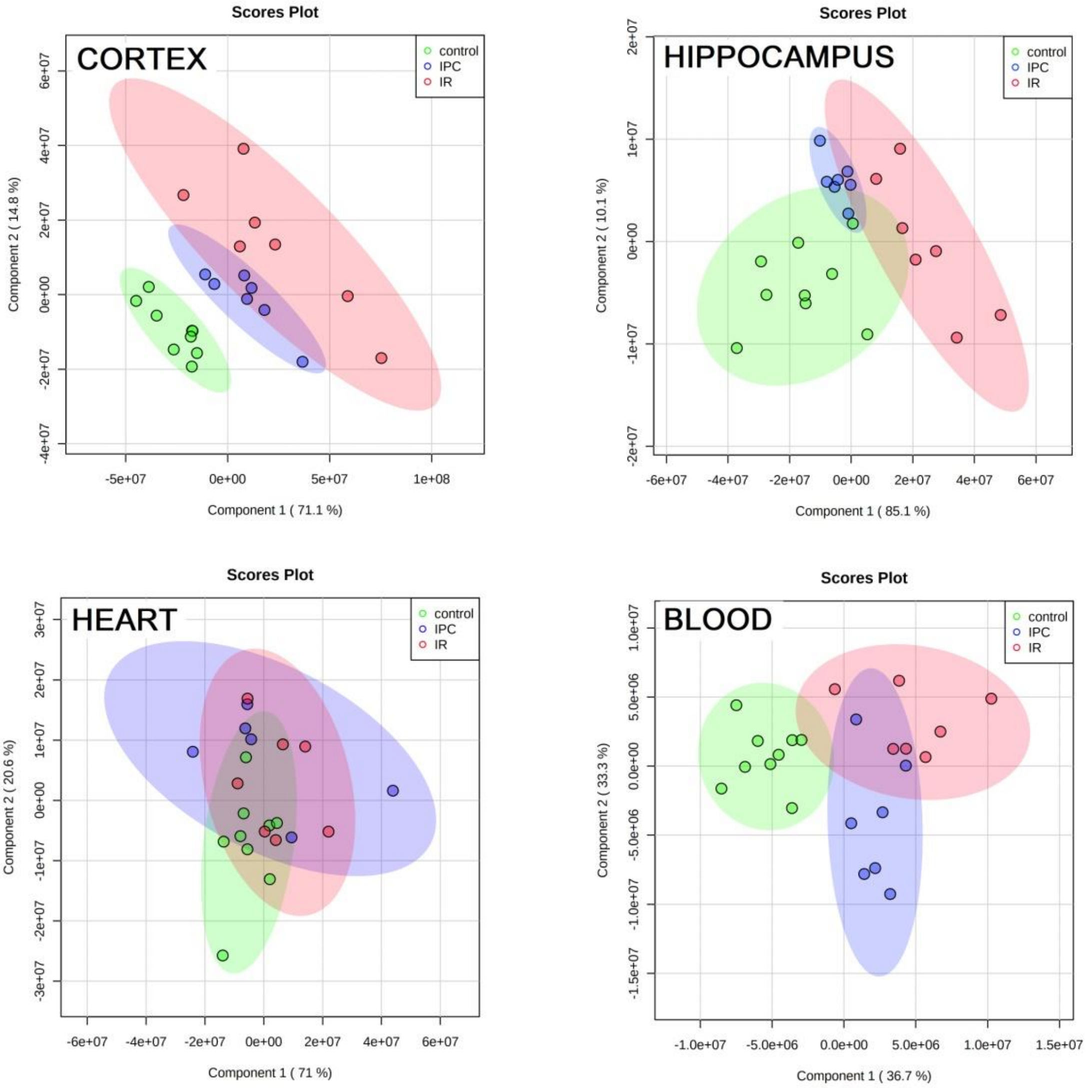

Figure S1. PLS-DA analyzes of system C-IR-IPC for cortex, hippocampus, heart and blood plasma in control, IR and IPC rats, as variables were used relative concentrations of metabolites determined by NMR.

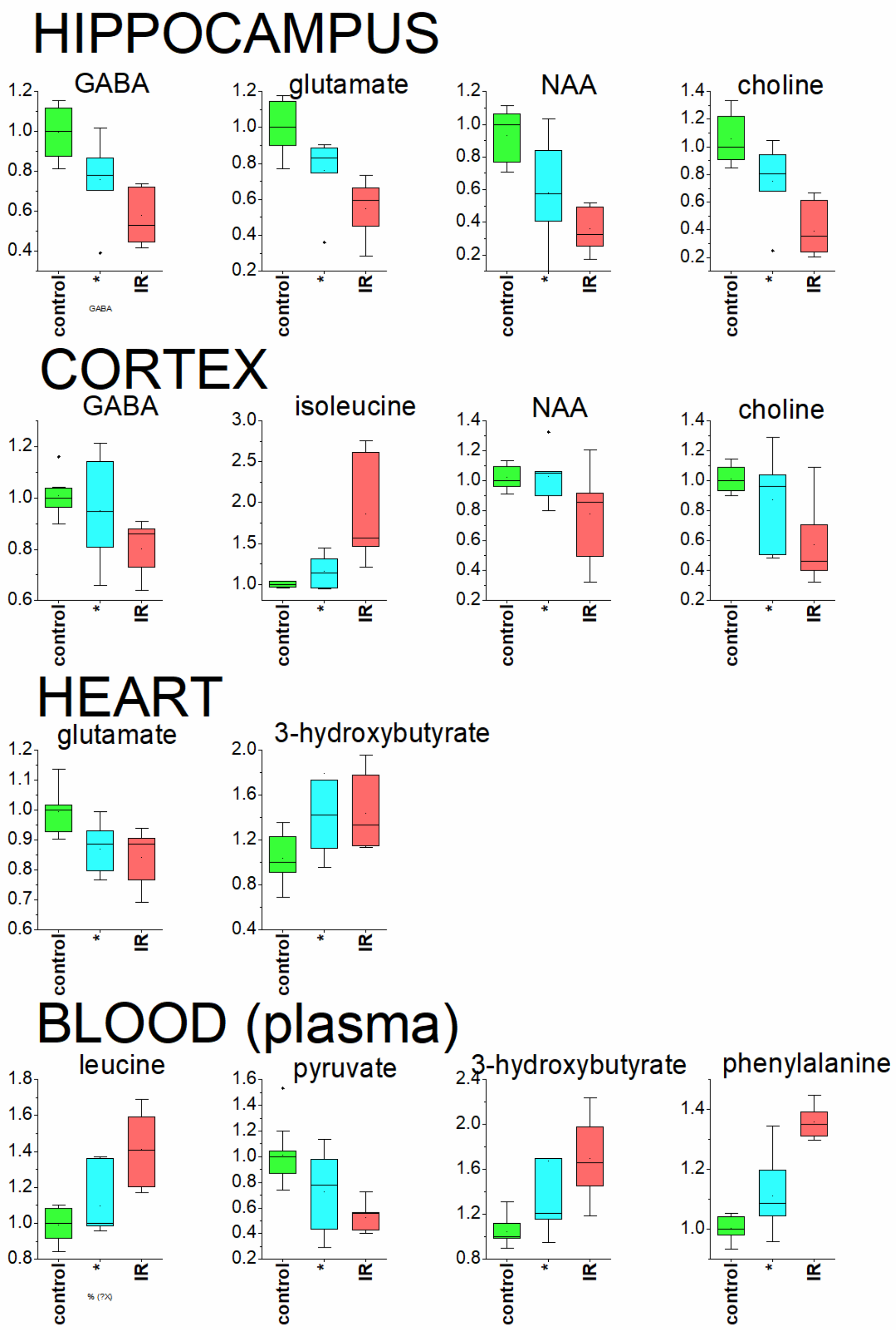

Figure S2. Relative levels of selected plasma metabolites in cortex, hippocampus and heart in ani-mals after detected and verified incomplete ischemia, * - rats not fulfilling criteria for ischemia completeness, IR - rats fulfilling criteria for ischemia completeness .

Table S1. Chemical shifts (in ppm), J couplings (in Hz) and multiplicities for the pool of metabolites identified in tissue extracts and deproteinated plasma by NMR. The proton NMR chemical shifts are reported relative to TMS-d_4 signal which was assigned a chemical shift of 0.000 ppm. +-metabolite found, - -metabolite not found, or assignment of peaks was not unambiguos Chemical shifts in particular material may differ from values listed, however the difference did not exceed 0.003 ppm.

metabolite	blood plasma	cortex	hippocampus	heart	NMR peak assignment, confirmed by jres and cosy
**lactate**	+	+	+	+	1.329 (d; J = 6.97), 4.116 (q; J = 6.97)
**glutamine**	+	+	+	+	2.1167 (m), 2.1534 (m), 2.441 (m), 2.477 (m), 3.766 (dd)
**histidine**	+	-	-	-	7.067 (s), 7.796 (s)
**isoleucine**	+	+	+	+	0.941 (t; J = 7.48), 1.0123 (d; J = 6.99), 3.678 (d; J = 4.17)
**leucine**	+	+	+	+	0.958 (d; J = 6.23), 0.969d (d; J = 6.05), 1.679 (m), 1.720 (m), 1.749 (m)
**lysine**	+	-	-	-	1.457 (m), 1.496 (m), 1.734 (m), 1.885 (m), 1.9255(m), 3.030 (t; J=7.63)
**phenylalanine**	+	+	+	+	3.126 (m), 3.284 (m), 7.3403 (d; J=7.46), 7.383 (t; J=7.36), 7.436 (t)
**threonine**	+	-	-	-	1.329 (d), 3.582 (d; J=4.93), 4.253 (m)
**tryptophan**	+	-	-	-	7.2087 (t), 7.292 (t), 7.334 (s), 7.556 (d), 7.743 (d; J = 8.02)
**tyrosine**	+	+	+	+	3.053 (dd), 3.193 (dd), 3.929 (dd), 6.906 (d; J=8.50), 7.201 (d; J=8.50)
**valine**	+	+	+	+	0.9936 (d; J=7.06), 1.044 (d; J=7.06), 2.273 (m), 3.607 (d; J=4.40)
**pyruvate**	+	-	-	-	2.376 (s)
**citrate**	+	-	-	-	2.5409 (d), 2.6717 (d)
**acetate**	+	-	-	-	1.9207 (s)
**alanine**	+	+	+	+	1.484 (d; J=7.30), 3.779 (q)
**glucose**	+	-	-	-	3.233 (m), 3.398 (m), 3.458 (m), 3.524 (dd), 3.782 (m), 3.824 (m), 3.889 (dd), 4.634 (d), 5.233 (d)
**3-hydroxybutyrate**	+	-	-	+	1.203(d; J=6.26), 2.307(dd; J= 14.36, 6.30), 2.393(dd; J= 14.36, 7.32), 4.138(m)
**2-oxoisocapronate (ketoleucine)**	+	-	-	-	0.943 (d; J=6.63), 2.113 (m), 2.612 (d; J=7.02)
**α-ketoisovalerate (2-ketovaline)**	+	-	-	-	1.11 (d; J=7.05), 3.011 (dq)
**3-methyl-2-oxo-valerate (2-ketoisoleucine)**	+	-	-	-	0.899 (t; J=7.52), 1.104 (d,; J=6.74)
**acetone**	+	-	-	-	2.236 (s)
**acetoacetate**	+	-	-	-	2.282(s), 3.427(s)
**formate**	+	-	-	-	8.461 (s)
**glutamate**	-	+	+	+	2.0055(m), 2.140(m), 2.346(m), 2.376(m), 3.756(dd; J = 7.18, 4.72)
**GABA (4-aminobutyrate)**	-	+	+	-	1.905(m), 2.3001(t; J=7.35), 3.025(t; J=7.58)
**succinate**	-	+	+	+	2.405(s)
**aspartate**	-	+	+	-	2.667(m), 2.820(m), 3.903(m)
**myo-Inositol**	-	+	+	-	3.282(t; J = 9.34), 3.534(dd). 3.629(t), 4.065(t; J = 2.91)
**creatine**	+	+	+	+	3.043(s), 3.928(t)
**taurine**	-	+	+	+	3.259(t; J = 6.57), 3.428(t; J = 6.61)
**niaccinamide**	-	+	+	+	7.600(dd, J = 7.90, 5.06), 8.252(dd, J = 7.98), 8.719(dd, J = 4.6, 1.12), 8.944(dd)
**fumarate**	-	+	+	+	6.519(s)
**inosine**	-	+	+	+	3.835(dd; J = 12.77, 4.03), 3.906(dd; J = 12.77, 3.09), 4.279(dd; J = 7.05, 3.08), 4.438(dd; J = 4.99, 3.95), 6.102(d; J = 5.67), 8.232(s), 8.337(s)
**ascorbate**	-	+	+	-	3.728(m), 3.760(m), 4.018(m), 4.508(d; J = 2.04)
**NAA (N-acetylaspartate)**	-	+	+	-	2.021(s), 2.494(m), 2.692(m), 4.367(m)
**choline**	-	+	+	+	3.203(s), 3.518(m), 4.057(m)
**o-phosphocholine**	-	+	+	+	3.221(s), 3.591(m), 4.172(m)

Table S2. Results from leave-one-out cross-validated PLS-DA statistical discrimination: R2, Q2 performance measured by acccuracy, * negative Q2 is a sign of overfitted model.

		number of components	accuracy	R2	Q2
**cortex**	IR/C	2	0.94	0.72	0.51
	IPC/C	5	1	0.99	0.9
	IR/IPC	2	1	0.92	0.85
	IR/IPC/C	3	0.95	0.74	0.48
**hippocampus**	IR/C	3	1	0.86	0.69
	IPC/C	3	1	0.91	0.77
	IR/IPC	5	1	0.92	-0.26*
	IR/IPC/C	4	0.91	0.89	0.65
**heart**	IR/C	3	0.75	0.75	0.08
	IPC/C	4	1	0.88	0.69
	IR/IPC	3	0.85	0.77	0.47
	IR/IPC/C	5	0.64	0.67	-0.25*
**blood**	IR/C	5	1	0.93	0.65
	IPC/C	2	0.94	0.82	0.6
	IR/IPC	3	0.93	0.88	0.44
	IR/IPC/C	4	0.87	0.86	0.68
